# A new set of ESTs and cDNA clones from full-length and normalized libraries for gene discovery and functional characterization in citrus

**DOI:** 10.1186/1471-2164-10-428

**Published:** 2009-09-11

**Authors:** M Carmen Marques, Hugo Alonso-Cantabrana, Javier Forment, Raquel Arribas, Santiago Alamar, Vicente Conejero, Miguel A Perez-Amador

**Affiliations:** 1 Instituto de Biología Molecular y Celular de Plantas (IBMCP), Universidad Politécnica de Valencia and Consejo Superior de Investigaciones Científicas (CSIC). Avenida de los Naranjos s/n, 46022 Valencia, Spain; 2 Current address : Centro de Investigación Príncipe Felipe. Avenida Autopista del Saler 16, 46012 Valencia, Spain; 3 Current address : Synergia Bionostra S.L. Ronda de Poniente 4, 28760 Tres Cantos, Madrid, Spain; 4 Current address : Instituto de Agroquímica y Tecnología de Alimentos (IATA), Consejo Superior de Investigaciones Científicas (CSIC), Burjassot, Valencia, Spain

## Abstract

**Background:**

Interpretation of ever-increasing raw sequence information generated by modern genome sequencing technologies faces multiple challenges, such as gene function analysis and genome annotation. Indeed, nearly 40% of genes in plants encode proteins of unknown function. Functional characterization of these genes is one of the main challenges in modern biology. In this regard, the availability of full-length cDNA clones may fill in the gap created between sequence information and biological knowledge. Full-length cDNA clones facilitate functional analysis of the corresponding genes enabling manipulation of their expression in heterologous systems and the generation of a variety of tagged versions of the native protein. In addition, the development of full-length cDNA sequences has the power to improve the quality of genome annotation.

**Results:**

We developed an integrated method to generate a new normalized EST collection enriched in full-length and rare transcripts of different citrus species from multiple tissues and developmental stages. We constructed a total of 15 cDNA libraries, from which we isolated 10,898 high-quality ESTs representing 6142 different genes. Percentages of redundancy and proportion of full-length clones range from 8 to 33, and 67 to 85, respectively, indicating good efficiency of the approach employed. The new EST collection adds 2113 new citrus ESTs, representing 1831 unigenes, to the collection of citrus genes available in the public databases. To facilitate functional analysis, cDNAs were introduced in a Gateway-based cloning vector for high-throughput functional analysis of genes *in planta*. Herein, we describe the technical methods used in the library construction, sequence analysis of clones and the overexpression of *CitrSEP*, a citrus homolog to the Arabidopsis *SEP3 *gene, in Arabidopsis as an example of a practical application of the engineered Gateway vector for functional analysis.

**Conclusion:**

The new EST collection denotes an important step towards the identification of all genes in the citrus genome. Furthermore, public availability of the cDNA clones generated in this study, and not only their sequence, enables testing of the biological function of the genes represented in the collection. Expression of the citrus *SEP3 *homologue, *CitrSEP*, in Arabidopsis results in early flowering, along with other phenotypes resembling the over-expression of the Arabidopsis *SEPALLATA *genes. Our findings suggest that the members of the *SEP *gene family play similar roles in these quite distant plant species.

## Background

Citrus is one of the most widespread fruit crops with great economic and health value [1]. But citrus is also one of the most difficult plants to improve through traditional breeding approaches due to undesirable reproductive traits and characteristics. These include degrees of sexual sterility and incompatibility, nucellar embryony (asexual seed production), extended juvenility, and large plant size, which affect cultural practice in the orchard. To overcome these drawbacks, new genomic approaches are being developed, including generation of linkage maps, markers, and EST collections, making possible physical and genetic mapping in citrus. Furthermore, an International Citrus Genomics Consortium (ICGC) has been initiated to generate the full-genome sequence of sweet orange (*Citrus sinensis*), as well as to sequence other citrus species and varieties [1]. Prior to the establishment of the ICGC, EST collections [2,3] have provided a first glimpse of the citrus genome. Over the years, several different groups have contributed to the generation of a total of over 230,000 citrus sequences currently deposited at the dbEST division of the GenBank. Among these, the Spanish Citrus Genomic Project (CFGP)  has made a significant contribution producing 25 standard cDNA libraries, an EST collection of 22,635 high-quality reads [4], and generating sequence data for over 54,000 ESTs from a normalized full-length cDNA library and 9 additional standard libraries [5]. EST sequencing along with other gene discovery methods, represent an important initial step towards functional characterization of the genes in the genome.

Many methods for the construction of cDNA libraries have been developed in recent years. Conventional cDNA library construction approaches, however, suffer from several major shortcomings. First, the majority of cDNA clones are not full-length, especially for mRNAs longer than 2 kb. This loss of 5'-terminal sequences is typically due to premature termination of reverse transcription or blunt-end polishing of cDNA ends prior to subcloning. As a result, cDNA 5' ends are significantly underrepresented in cDNA libraries. Second, an adaptor-mediated cloning process is still a common approach for cDNA library construction, leading to up to 20% of undesirable ligation by-products (chimeras) and inserts of non-mRNA origin (e.g., genomic DNA, mitochondrial DNA, ribosomal RNA, or adaptor dimers) [6]. In recent years, the annotation of genes has been greatly improved by the integration of full-length cDNAs produced by the community [7-10]. The importance of isolating full-length cDNA clones relies in the "value-added" features lacking in common ESTs. Full-length cDNAs define the limits of the transcriptional units and the coding region, and thus identify the immediate upstream basal promoter and enable sequence characterization of 5' and 3' untranslated regions (UTR). Furthermore, they provide a record of transcript diversity due to modifications of the primary pre-mRNA transcript, such as alternate promoter usage, alternative splicing, alternate polyadenylation, and RNA editing. On the other hand, cDNA libraries rich in full-length clones are a valuable tool for high-throughput gene function analysis [11].

A number of methods have been developed for cDNA library preparations enriched in full-length sequences [12-18] with most of them based on the mRNA cap structure [12,14,15]. These methods require high quantities of starting material (20-100 μg of RNA) and complicated, multi-step manipulations of the cap structure of mRNA and cDNA intermediates, which often result in degradation of mRNA and isolation of short clones, no longer than 1.5 kb-long [6]. By improving one of these methods, Suzuki and co-workers obtained an average size of full-length clones of 1.9 kb from lower amounts of starting mRNA (5-10 μg) [16,19]. The recently described SMART™ method exploits two intrinsic properties of Moloney murine leukaemia virus (MMLV) reverse transcriptase, reverse transcription and template switching, and thus yields larger average ORF size [6,20]. Moreover, directional cloning has been implemented by using *Sfi*I endonuclease, whose variable target sequence allows for designing adaptors with non-complementary ends, thus avoiding their concatenation. As the *Sfi*I recognition sequence is very rare in eukaryotic genomes, the use of *Sfi*I also eliminates the need for methylation during cDNA synthesis [21].

The differential abundance of various transcripts in any particular cell type is a well recognized obstacle for the efficient high-throughput analysis of cDNA libraries [18]. Usually 10-20 abundant genes (present at several thousand mRNA copies per cell) account for at least 20% of the cellular mRNA mass, while several thousand of rare genes (each represented less than 10 mRNA copies per cell) may account for 20-40% of the mRNA mass [18]. Hence, straightforward random sequencing of clones from standard cDNA libraries is inefficient for discovering rare transcripts, owing to the repeated occurrence of intermediate and highly abundant cDNAs. Decreasing the prevalence of clones representing abundant transcripts by normalizing the cDNA before sequencing may significantly increase the efficiency of random sequencing and is essential for rare gene discovery [22,23]. The normalization process generally utilizes second-order reaction kinetics of re-association of denatured DNA, so that relative transcript concentrations within the remaining single-stranded cDNA fraction are equalized to a considerable extent. Most of the normalization methods described differ in the process of isolation of the single-stranded (ss) and double-stranded (ds) cDNA fractions but typically employ one or more of the following: hydroxylapatite columns, magnetic beads, digestion of the ds-molecules by restriction endonucleases or amplification of the ss fraction using suppression PCR [24]. One of the recently described method takes advantage of the properties of a particular nuclease, the DSN from the Kamchatka crab, to specifically cleave ds-DNA in both DNA-DNA and DNA-RNA duplexes, allowing for the separation of the normalized ss-fraction [24-26]. DSN is a thermo-stable enzyme active at 70°C, and therefore, the degradation of the ds-fraction is carried out at the same temperature as the re-naturation of cDNA. This helps to avoid a non-specific hybridization of cDNA during the DSN treatment and, hence, minimizes the loss of transcripts prone to the formation of secondary structures. The remaining normalized ss-fraction is then amplified using PCR [24,25]. Suitable cDNA for this procedure should contain known flanking sequences for subsequent PCR amplification. Furthermore, to avoid the preference towards shorter products, a process of regulation of average length is recommended [27]. The use of this method requires that the adapter sequences form inverted terminal repeats [25,27].

On the other hand, moving beyond gene discovery to understanding gene function is facilitated by the ability to easily express proteins in both homologous and heterologous biological contexts. The functional analysis of genes typically requires each ORF to be over-expressed or silenced, the purification of the expressed protein, production of antibodies, analysis of phenotypes, determination of intracellular localization, and analysis of interactions with other proteins. This entails engineering of multiple expression constructs for every gene of interest, which is time-consuming and laborious when using traditional ligase-mediated cloning, posing a technical barrier for high-throughput functional genomics or proteomics projects [28]. Hartley and co-workers described a method called recombinational cloning that exploits the accurate and site-specific recombination system utilized by bacteriophage lambda in order to shuttle sequences between plasmids bearing compatible recombination sites [29]. This technology, commercially termed Gateway™ (Invitrogen), bypasses the need for traditional ligase-mediated cloning, provides high specificity and activity, while maintaining orientation of the transferred DNA segment and yielding a high proportion of desired clones [28-32].

We took advantage of the SMART™ protocol, the DSN nuclease, and the Gateway technology to maximize acquisition of full-length and rarely-expressed cDNAs (from various tissues and under different conditions), ready to use for functional analysis purposes. Herein, we describe the methods employed to generate a new citrus cDNA collection, giving rise to a new set of ESTs and unigenes. As a direct example of the practical application of our collection, we carried out the overexpression of a full-length cDNA clone for the *CitrSEP *gene in Arabidopsis. The resulting transgenic plants showed early flowering and curly leaves, phenotypes that are consistent with a role of *CitrSEP *as a *bona fide *citrus ortholog of the Arabidopsis *SEP3 *gene. The protocol utilized in this study has thus been proven to be successful for generating new EST collections to improve gene annotation and gene discovery by functional characterization.

## Results and Discussion

### Full-length and normalized cDNA libraries

The Spanish Citrus Genomic Project (CFGP) was initiated with the purpose of functional characterization of citrus genes. It has generated an EST collection from standard cDNA libraries covering a wide range of tissues and developmental stages, as well as biotic and abiotic stress conditions [4]. In addition, cDNA microarray platforms have also been created [4,33]. In this work, we wanted to enrich the EST collection with the addition of a significant number of full-length cDNA clones. With this objective we decided to use the SMART™ method, as it allowed us to obtain large ESTs with few steps and from small quantities of starting RNA. Furthermore, the subcloning advantages of the Gateway system, which allows a captured target sequence to be easily shuttled into a variety of destination vectors, provides great advantages for future functional analyses of the isolated gene. For that reason, we modified the pENTR1A vector in order to make it suitable for the directional cloning of citrus cDNAs. A detailed description of the construction of the Gateway-based pENTR-*Sfi*I is shown in Methods and Additional File 1. The engineered pENTR-*Sfi*I cloning vector proved to be a useful tool for the construction of the cDNA libraries as it rendered a very high cloning efficiency and almost all of the clones generated contained cDNA inserts introduced in the direct orientation as single inserts (data not shown).

We constructed a total of 15 cDNA libraries (Table 1) using the SMART™ method and the engineered Gateway-based pENTR-*Sfi*I cloning vector. In order to increase the proportion of full-length clones, we also performed a size selection of the cDNAs, by removing those cDNAs that were shorter than 1 kb, presumably corresponding to partial cDNAs and/or cDNAs that could have already been isolated in our standard cDNA libraries constructed previously [4]. Since EST collections are hampered by the presence of cDNA clones corresponding to highly expressed genes, which limit the probability of isolation of new gene sequences, we decided to include a normalization step in the construction of 4 of the cDNA libraries to isolate ESTs corresponding to rare or low-expressed genes. In order to get wide transcriptome coverage, multiple libraries were constructed from leaves, roots, shoots, flowers, ovaries and fruits of different citrus species under different stress or developmental conditions (Table 1).

**Table 1:** Plant material used in each individual cDNA library, either full-length or full-length and normalized.

**Library**	**Species**	**Treatment**	**Tissue**	**Developmental** **stage**
**Full-length enriched libraries**				
AbioticL1	*C clementina*	Several abiotic stresses*	Leaves	Adult
AbioticR1	*C reshni*	Several abiotic stresses*	Roots	Young
BiotPhyR1	*C aurantium*	Infection with the oomycete *Phytophthora citrophthora*	Roots	Young
CTVMacrop1	*C macrophylla*	Infection with the Citrus tristeza virus (CTV)	Leaves	Adult
CTVClemen1	*C clementina*	Infection with the Citrus tristeza virus (CTV)	Shoot	Adult
CEVdCidro1	*C medica*	Infection with the Citrus exocortis viroid (CEVd)	Leaves	Adult
HSVdMacro1	*C macrophylla*	Infection with HSVd	Leaves	Adult
DevOvary1	*C clementina*	Normal culture conditions	Ovaries	Adult
RVDevelop1	*C clementina*	Normal culture conditions	Leaves, flowers, ovaries and apical meristems	Adult
PostharvC1	*C clementina*	Cold stress over harvested fruit	Mature fruit (flavedo)	Adult
PostharvP1	*C clementina*	Infection with the fungus *Penicillium digitatum*	Mature fruit (flavedo and albedo)	Adult

**Normalized and full-length enriched libraries**				
RVDevelopN	*C clementina*	Normal culture conditions	Leaves, flowers, ovaries and apical meristems	Adult
StrClemenN	*C clementina*	Several abiotic stresses* Infection with the Citrus tristeza virus (CTV)	Abiotic stress: Leaves Biotic stress: Shoots	Adult
StrCleopN	*C reshni*	Several abiotic stresses* Infection with the oomycete *Phytophthora citrophthora*	Roots	Young
PostharveN	*C clementina*	Cold stress over harvested fruit Infection with the fungus *Penicillium digitatum*	Abiotic stress: Fruits (flavedo) Biotic stress: Fruits (flavedo + albedo).	Adult

* water stress, salt stress, progressive salt stress, and iron chlorosis.

### EST collection

A total number of 11,968 independent cDNA clones were randomly isolated from the 15 cDNA libraries and single-pass sequenced from the 5' end of the clone to generate the EST collection (Table 2). After low-quality and vector trimming of raw sequences, a total number of 10,898 high-quality ESTs longer than 100 bp were obtained. High-quality sequences were deposited in the dbEST division of GenBank (accession numbers FC868488-FC870221, FC873874-FC876453, FC877373-FC877779, FC920173-FC921754, FC923090-FC924869, and FC929840-FC932655). Size distribution analysis showed that, after trimming of vector and poor quality sequences, most ESTs (89%) were longer than 600 bp, with an average sequence length of 673 nucleotides (Additional File 2A).

**Table 2:** Characterization of cDNA citrus libraries.

**Library**	**Clones**	**High** **quality** **ESTs**	**Mean** **EST** **length**	**Singletons**	**Contigs**	**Contigs** **with** **2-3** **reads**	**Unigenes**	**Redundancy** **(%)**	**Library-** **specific** **unigenes**	**Novelty** **(%)**	**Full-** **length** **clones**	**ESTs** **with** **orthologue**	**Fullness**	**Full-** **length** **library-** **specific** **unigenes**
**Full-length enriched libraries**														
AbioticL1	960	831 (87)	673	125	498	481 (97)	623	25	139	22	613	794 (96)	77	46
AbioticR1	960	903 (94)	667	157	531	514 (97)	688	24	168	24	698	853 (94)	82	49
BiotPhyR1	960	919 (96)	681	364	401	389 (97)	765	17	407	53	515	752 (82)	68	31
CTVMacrop1	480	447 (93)	651	133	214	205 (96)	347	22	147	42	350	412 (92)	85	58
CTVClemen1	480	453 (94)	682	62	327	324 (99)	389	14	63	16	329	426 (94)	77	48
CEVdCidro1	480	465 (97)	693	111	220	214 (97)	331	29	152	46	330	446 (96)	74	59
HSVdMacro1	480	406 (85)	654	116	209	201 (96)	325	20	128	39	281	373 (92)	75	62
DevOvary1	384	296 (77)	637	27	192	185 (96)	219	26	29	13	251	291 (98)	86	69
RVDevelop1	960	759 (79)	669	60	451	433 (96)	511	33	65	13	612	737 (97)	83	46
PostharvC1	960	917 (96)	697	134	582	565 (97)	716	22	137	19	706	886 (97)	80	44
PostharvP1	1056	1008 (95)	684	161	640	624 (98)	801	21	169	21	662	966 (96)	69	44
**TOTAL**	8160	7404 (91)		1450	2914		4364		1604	37	5347	6936 (94)	78	556

														

**Normalized and full-length enriched libraries**														
RVDevelopN	960	823 (86)	640	182	543	540 (99)	725	12	192	26	560	755 (92)	74	44
StrClemenN	960	866 (90)	658	186	567	563 (99)	753	13	203	27	544	810 (94)	67	45
StrCleopN	960	914 (95)	693	246	594	594 (100)	840	8	254	30	655	866 (95)	76	51
PostharveN	928	891 (96)	684	176	561	552 (98)	737	17	185	25	636	840 (94)	76	41
**TOTAL**	3808	3494 (92)		790	1882		2672		834	31	2395	3271 (94)	73	181

														

**BIG TOTAL**	11968	10898 (91)	673	2240	3902		6142		2438	40	7742	10207 (94)	76	737

Since new sequences are to be incorporated with the previous CFGP collection, EST assembly was carried out together with the other ESTs obtained in the CFGP. It revealed that ESTs from our full-length libraries could be clustered in 2240 singletons and 3902 contigs, yielding a total number of 6142 putative unique transcripts or unigenes (Table 2). This number of unigenes is probably an overestimation of the number of unique transcripts isolated, as failure to assemble ESTs from a single transcript can result from alternate splicing, sequence polymorphisms, sequencing errors, and non-overlapping ESTs. To estimate this internal redundancy, the 6142 putative unigenes were compared with each other using BLASTN. Sequences with at least 90% nucleotide identity over a minimum of 300 bp covering at least 75% of one sequence were assumed to be derived from the same transcript or from different transcripts coming from the same gene (e.g., alternative splicing and polyadenylation), and were therefore clustered in super-contigs (see Methods). This analysis resulted in 4691 unigenes remaining as singletons and 1451 unigenes being clustered in 666 super-contigs, indicating that the minimal number of identified expressed genes was 5357.

The number of ESTs per contig ranged from 2 (1016 contigs) to 78 (one contig, corresponding to a lectin-related protein), while most contigs (83%) contained 4 or fewer ESTs, and only 15 contigs (0.8% of total) included more than 20 EST sequences (Additional File 2B). ESTs that constitute large clusters partially overlap, thus allowing the reconstitution of the full-length sequence of genes without having to use expensive and labor-intensive primer walking sequencing. These redundant sequences are also a source of single nucleotide polymorphisms (SNPs) for molecular marker development, and 233 contigs were found to have putative SNPs (data not shown).

### Improvement of the previous CFGP citrus EST collection

We estimated the contribution of our new EST collection to the previous CFGP EST collection by calculating the redundancy and novelty of the full-length libraries constructed in this study. Table 2 shows the distribution per library of the number of ESTs, singletons, contigs, and unigenes, as well as the absolute and relative values for redundancy and novelty. Since we are interested in the optimization of the sequencing effort for the entire project, redundancy for each library was calculated as the percentage of ESTs in this library that correspond to unigenes already obtained in the whole project. Although this number is necessarily higher than redundancy within the library, most libraries (11 out 15) had a level of redundancy below 25%, while in the most redundant library it was higher (33%) but still acceptable. On the other hand, novelty was calculated as the percentage of unigenes in each library that have been isolated only from that particular library (unique unigenes). This number represents the level of uniqueness of the library, which can also be considered as an estimation of the capacity of the library to provide new genes to the collection, or gene discovery. The full-length libraries constructed in this study have novelty range of 13-53%, with only 4 libraries having a novelty lower than 20% and three of them exceeding 40% (Table 2). The contribution of the full-length libraries to the whole citrus EST collection is therefore indicated by the low levels of redundancy and high percentages of novelty of these libraries. A major advantage of EST sequencing from multiple libraries is that it increases the possibility of identifying genes that are putatively transcribed specifically within a certain tissue, during a particular developmental phase, or under some environmental conditions. Our analysis indicates that 2438 unigenes (24% of 6142) are specific for the newly constructed libraries, despite the presence of 85,965 ESTs in the entire CFGP collection, suggesting the utility of these new libraries.

We also compared the new citrus ESTs with the ESTs and unigenes already available in the Citrus HarvEST database, which contains 229,570 ESTs. We estimate that the new EST collection adds 2113 new citrus ESTs representing 1831 unigenes (Additional File 3). In other words, around 20% of ESTs and 30% of unigenes generated were not previously available in the public database and therefore represent new genes.

### Genomic coverage of the unigenes identified

We estimated the genomic coverage of the unigenes identified in the present work by comparing their functional distribution to that of a full sequenced plant genome. Table 3 shows the distribution of the citrus unigenes and Arabidopsis genome along the main GO functional categories in the 'Biological Process' ontology. The distribution along the main functional categories in the three different GO ontologies is showed in the Additional File 4. These results show that the different unigenes obtained are involved in many different categories covering virtually every aspect of plant biology and offer a good representation of the citrus genome.

**Table 3:** Distribution of citrus and Arabidopsis genes according to the GO categories of Biological process.

**GO category**	**Citrus**	**TAIR**
transport	8.41	4.43
protein modification process	6.47	3.50
translation	5.49	3.07
carbohydrate metabolic process	4.58	2.00
catabolic process	4.28	1.50
transcription	4.08	4.21
amino acid and derivative metabolic process	3.94	1.21
response to stress	3.81	4.77
cellular component organization and biogenesis	2.95	3.05
lipid metabolic process	2.36	1.72
cell communication	2.29	2.74
generation of precursor metabolites and energy	2.24	0.52
signal transduction	2.21	2.41
secondary metabolic process	1.92	0.83
response to abiotic stimulus	1.55	2.79
photosynthesis	1.06	0.35
response to endogenous stimulus	0.93	1.90
cell death	0.71	0.33
death	0.71	0.33
response to external stimulus	0.71	0.67
DNA metabolic process	0.57	0.78
response to biotic stimulus	0.49	1.50
reproduction	0.34	2.38
cellular homeostasis	0.30	0.38
anatomical structure morphogenesis	0.25	0.97
growth	0.17	0.63
post-embryonic development	0.15	1.20
cell growth	0.12	0.52
embryonic development	0.10	1.23
cell cycle	0.07	0.50
flower development	0.07	0.64
ripening	0.05	0.01
regulation of gene expression, epigenetic	0.02	0.35
Abscission	0.00	0.03

Citrus genes are those reported here and Arabidopsis genes are those from TAIR. Data are shown as percentages.

### Full-length cDNA clones

Since libraries were constructed using oligo-dT for cDNA synthesis and inserts were 5'-end sequenced, we estimated the number of putative full-length cDNA clones generated by calculating the number of clones that had BLASTX matches to proteins of the reference plant Arabidopsis that included the first amino acid of the Arabidopsis protein (Table 2). A total of 3304 unigenes were identified as having putative full-length clones by this method. In most of these cases, the unigene sequence also contains additional sequence upstream of the start codon in the alignment, possibly corresponding to 5'-UTR. These putative full-length unigenes represent 63% of the total number of unigenes having matches with Arabidopsis proteins (5248 unigenes), indicating that about 3870 unigenes (63% of the total number of unigenes with some of the clones coming from the libraries constructed herein) are expected to have at least one full-length cDNA clone among their components. However, identification of full-length clones using this approach is a relatively crude method, and these values must be only taken as a rough estimate.

We next investigated the efficiency of the library construction protocol with regard to the enrichment in full-length clones. The comparison with the results for the libraries not enriched in full-length clones clearly indicates that full-length libraries described here produced a considerably higher percentage of full-length unigenes (>40% in 14 out of 15 libraries; two of them >60%) (Table 2) than the other standard libraries (none above 32%) [4]. Furthermore, full-length estimation by ESTs instead of unigene sequences emphasized the benefits of the full-length enrichment protocol. While the putative full-length ESTs in other standard libraries account for 30% to 69% (44% on average, subtraction libraries including a cDNA fragmentation step in their protocol were not considered, data not shown), those in our full-length libraries comprise 67% to 86% (77% on average) (Table 2).

### Efficiency of cDNA libraries normalization

Most normalization strategies are based on the reaction kinetics of re-association of denatured DNA. However, not all of them are amenable to full-length cDNA approaches. The most widely used technology for normalization and substraction in large-scale gene discovery relies on the re-association of nucleic acids in amplified plasmid libraries [22,23] and consequently suffers from cDNA-size cloning bias that can lead to under-representation of long cDNAs. To avoid the problems related to the amplification of libraries, Carninci et al. [18] developed a method to normalize and subtract cDNA before cloning. In that method, a biotinylated driver (usually an aliquot of the mRNA initially used for the cDNA library preparation) is employed to detect and eliminate abundant cDNAs. However, this method has several drawbacks as it requires high quantities of starting poly(A^+^) RNA and is time-consuming [25].

Among the different methods employed to separate the normalized ss-cDNA fraction we chose normalization with the thermostable DSN nuclease [24,25]. It displays a strong preference for cleaving double-stranded DNA and DNA in DNA-RNA hybrid duplexes compared with ss-DNA and ss-RNA, irrespective of sequence length. Due to the thermostable properties of the DSN enzyme, the degradation of the double-stranded fraction is carried out at the same temperature as the re-naturation of cDNA. This helps to avoid a non-specific hybridization of cDNA during the DSN treatment and, hence, the loss of transcripts which are prone to the formation of secondary structures.

On the other hand, each cDNA population requires a specific normalization treatment (different quantities of DSN enzyme). Those normalized cDNAs were then subjected to amplification by PCR in order to identify the population that had undergone the best normalization. Aliquots of the PCR reaction were extracted at different amplification cycles and tested by electrophoresis to identify the samples showing a good normalization profile (Additional File 5). The profile of an efficiently normalized and amplified cDNA was one whose overall signal intensity of a smear (at its plateau) was similar to that shown for the control, but does not contain distinguishable bands nor shows smear in the high-molecular-weight region of the gel (> 4.5 kb). Finally, a virtual northern [34] was carried out to estimate the relative concentration of a highly abundant clone in both the non-normalized and the normalized cDNA populations obtained from the second run of amplification (Additional File 5C and 5D). The clone C32009H03 from the contig aCL11contig11, whose consensus sequence shows similarity with the family of lectin-related protein kinases, was selected as probe because it was present at high copy number in the equivalent full-length non-normalized cDNA library RVDevelop1, revealing the high expression of the corresponding citrus gene. Equal amounts of normalized and non-normalized cDNA samples were electrophoresed and transferred to a nitrocellulose membrane. The reduction in the abundance of the frequent clone, used as a radioactive probe, in the normalized sample became evident since much lower signal intensity was detected when compared to that observed in the non-normalized cDNA.

In summary, 4 out of the 15 full-length cDNA libraries were normalized using this approach. Overall, the redundancy of these libraries is lower than that of non-normalized libraries (Table 2). Furthermore, when the distribution of the number of ESTs per contig is compared, normalized libraries show a higher proportion of contigs with a low number of ESTs and a lower proportion of contigs represented by a high number of ESTs (Additional File 3). Finally, the new citrus ESTs collection added almost 2000 new genes not previously represented in the Citrus HarvEST database, which already contains 229,570 ESTs. These results show a good efficiency for the normalization step and demonstrate the convenience of this approach to improve the efficacy of the sequencing effort at isolating new and/or low expressed transcripts in the EST collections.

### Functional analysis of citrus genes by transformation of Arabidopsis plants

Arabidopsis is the reference system for plant biologists [35] due to several characteristics, such as the availably of its full-genome sequence and simplicity of transformation, which makes it an excellent system for functional analysis of heterologous genes. For the ectopic expression of citrus genes in Arabidopsis, we chose the destination vector pMDC32 designed by Curtis et al. [31]. The backbone of this vector is derived from the pCambia series of binary vectors for *Agrobacterium*-mediated plant transformation. The Gateway cassette is adjacent to the dual 35S CaMV promoter, so the expression of the heterologous genes is under the control of this strong promoter.

To validate the system implemented in our cDNA collection for rapid functional characterization of citrus genes, we selected a citrus member of the *SEPALLATA *gene family of transcription factors with MADS-box domain. Plant MADS-box gene family participates in the determination of floral organ identity. Among them, the *SEPALLATA *are a class of MAD-box genes with a role as floral homeotic genes that are required for the development of petals, stamens and carpels [36]. In Arabidopsis, the *SEPALLATA *gene family is composed of four members (*SEP1 *to *SEP4*). Triple mutant Arabidopsis plants lacking the activity of three *SEP1*/2/3 genes produce flowers in which all organs develop as sepals [36]. Moreover, over-expression of these genes provokes alterations in organ identity as well as early flowering and curly leaves [37]. In addition, other *SEPALLATA *genes from different species have been expressed in Arabidopsis. For example, overexpression of either lily *LMADS3 *or wheat *TaMADS1*, both orthologs of Arabidopsis *SEP3*, causes early flowering after producing only four or five curly rosette leaves [38,39]. Thus *SEP *gene function is easy to score by ectopic expression in a validated heterologous system, like Arabidopsis.

**Figure 1:**
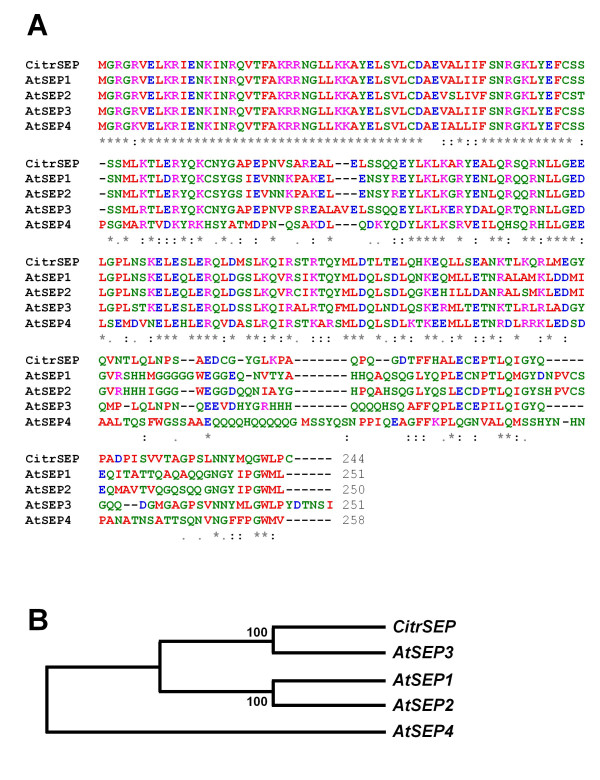
**Sequence comparison of *CitrSEP *and Arabidopsis orthologues**. **A**, Protein sequence alignment of *CitrSEP *with Arabidopsis orthologues *SEP1 *(*At5g15800*), *SEP2 *(*At3g02310*), *SEP3 *(*At1g24260*), and *SEP4 *(*At2g03710*). **B**, Phylogenetical tree of the aminoacid sequences of *CitrSEP *and the four Arabidopsis *SEP *genes, constructed with the ClustalW program .

To express a citrus *SEP *gene in Arabidopsis, we first searched our citrus database for a full-length clone showing the highest homology to *SEPALLATA *genes. In alignment assays, the clone C32006D10, that had been isolated from the cDNA library RVDevelop1, showed a high degree of sequence identity (75%) with the Arabidopsis gene *SEP3*. The C32006D10 clone was completely sequenced. It corresponds to a 999-nt transcript, with a deduced protein sequence of 244 amino acids with a molecular weight of 27.8 kDa (Figure 1 and Additional File 6). Aminoacid sequence also showed a very high sequence similarity to Arabidopsis *SEP *proteins (Figure 1A), indicating that the C32006D10 clone corresponds to the citrus orthologs of the Arabidopsis *SEP3 *(Figure 1B). Thus, the corresponding gene was named *CitrSEP*. More recently, five citrus *SEP *genes (*CitMADS1/3/5/6/8*) have been isolated and characterized [40]. Although *CitrSEP *is almost identical to *CitMADS3*, they differ in the first 4 aminoacid residues (data not shown), with the amino-end sequence of *CitrSEP *(MGRG-) being identical to all Arabidopsis *SEP *genes (Figure 1A), unlike the distinct N-terminal sequence of *CitMADS3*, MARGG-. Thus, *CitrSEP *and *CitMADS3 *appear to correspond to different but highly related citrus genes.

**Figure 2:**
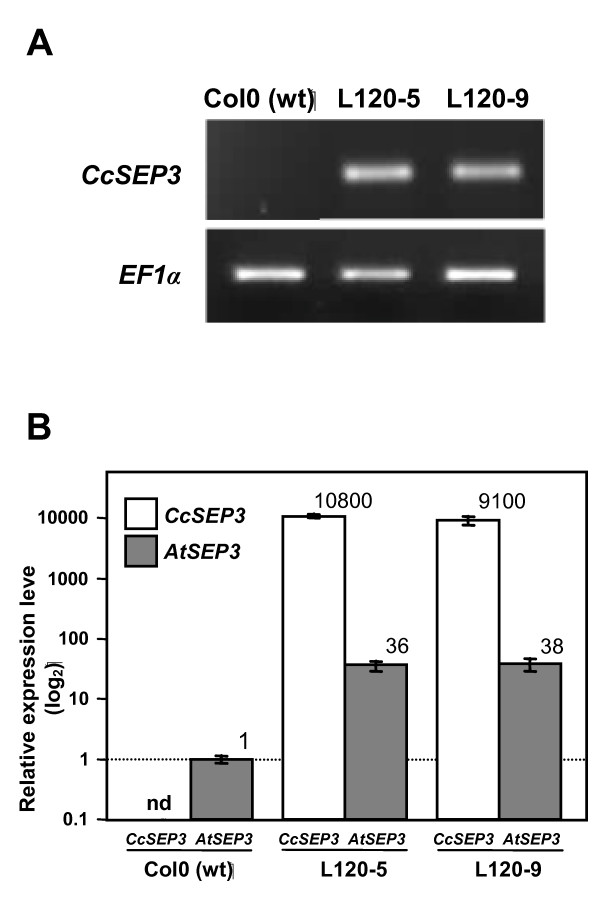
**Expression analysis of transgenic Arabidopsis plants that overexpress the *CitrSEP *gene**. **A**, Semi-quantitative RT-PCR analysis of 12-day old untransformed Col-0 and transgenic lines L120-5 and L120-9. **B**, qRT-PCR of *CitrSEP *and endogenous *SEP3 *in transgenic lines. Expression was normalized to the expression of the constitutive *EF-1-α *gene, and then to the expression in Col-0 plants. For normalization purposes, the detection level of our qRT-PCR analysis was used as an estimate of *CitrSEP *expression in Col-0. Expression level is indicated in the plot. nd, not detected.

**Figure 3:**
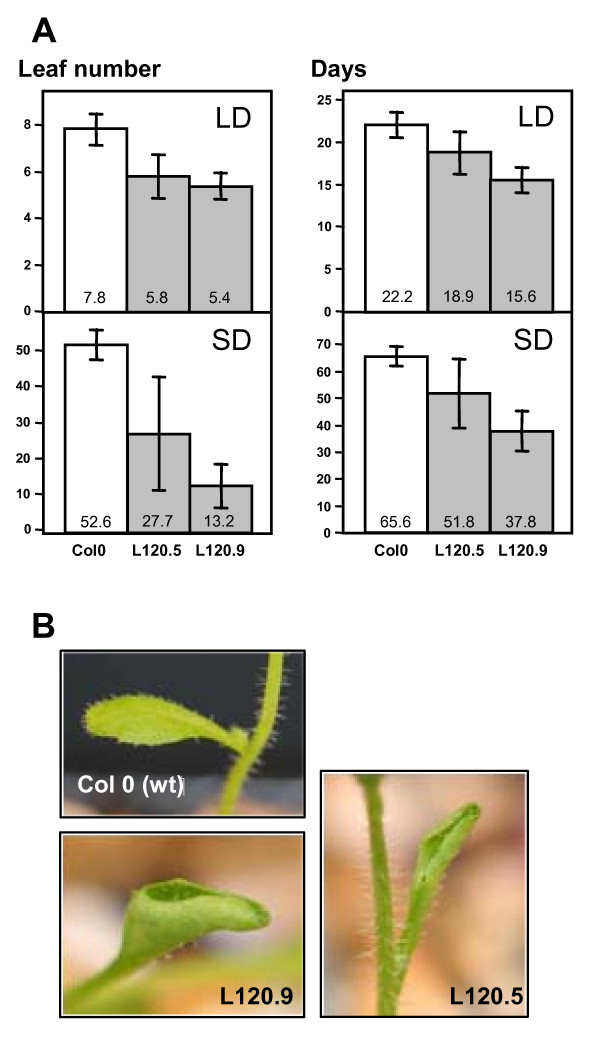
**Overexpression of *CitrSEP *provokes early flowering in transgenic lines**. **A**, quantification of flowering time in untransformed Col-0 and transgenic L120-5 and L120-9 lines. Leaf number (left panels) or days (right panels) upon flowering are as indicated. **B**, Images of the curly leaf phenotype in transgenic lines.

The *CitrSEP *cDNA clone was then transferred to the destination vector pMDC32 by means of LR clonase reaction as detailed in Methods. This clone was introduced in Arabidopsis, and several hygromycin-resistant homozygous lines were selected. Figure 2 shows the expression of *CitrSEP *gene in transgenic lines L120-5 and L120-9, which displayed the highest expression levels and were thus selected for further analysis. Next, we tested the expression of the endogenous *SEP *genes, as well as *CitrSEP*, by qRT-PCR with gene-specific primers. Interestingly, along with the overexpression of the *CitrSEP *gene, the endogenous *SEP3 *was also strongly upregulated in these lines (Figure 2), while the other three *SEP *genes were also affected but to a lesser extent (Additional File 7), suggesting a positive feedback on its regulation whose characterization goes beyond the scope of this work. Recently, it has been reported that SEP3 interacts with other MADS-box proteins, including other SEP, to form multimeric protein complexes, suggesting a role as mediator of higher-order complex formation [41]. Transgenic plants were grown to maturity and their phenotypes were scored. Both lines bolted earlier than control plants, both in long and short days (Figure 3A). In addition, leaves of transgenic lines showed curly leaves (Figure 3B). All of these phenotypes were indistinguishable from those already reported for the overexpression of *SEP *genes in Arabidopsis, suggesting that *CitrSEP *is indeed a functional homolog of *SEP3 *in citrus.

The availability of an easy and efficient transfer method enabling subcloning of full-length cDNAs into expression vectors allows for the rapid analysis of gene function in citrus. Although we fully sequenced the *CitrSEP *clone as a proof of concept, the system implemented in our EST collection bypasses the need for cDNA sequencing, as clones can be rapidly transferred regardless of their sequence information, without restriction enzyme digestion utilized in classical cloning methods. We believe that this collection can provide an enormous advantage for gene validation in citrus. Furthermore, the enrichment of full-length clones facilitates the task of genome annotation once the whole-genome sequence becomes available in the near future. The low percentage of redundancy among the clones isolated so far guarantees that many other genes can be successfully identified in new rounds of clone selection and sequencing from these libraries.

## Conclusion

We isolated a new set of 10,898 high-quality ESTs representing 6142 different genes from 15 normalized and non-normalized cDNA libraries, using an integrated methodology. These sequences provided 2113 new citrus ESTs, representing 1831 unigenes, to the collection of citrus genes available in the public databases. Our collection shows enhanced enrichment for full-length transcripts thus facilitating downstream functional analysis of newly discovered genes. For this purpose, citrus cDNAs were introduced in a Gateway-based cloning vector for rapid high-throughput functional analysis of genes *in planta*. As a proof of concept of the genomic tool generated, expression in Arabidopsis of the citrus *SEP3 *homologue, *CitrSEP*, was shown to lead to early flowering, along with other phenotypes mimicking the over-expression of the Arabidopsis *SEPALLATA *genes. These findings suggest that the *SEP *gene family performs similar roles in distantly related plant species, citrus and Arabidopsis, and demonstrate the utility of the new full-length cDNA clone collection for the analysis of gene function.

## Methods

### Plant material and treatments

Citrus leaves, shoots, roots, developing flowers, and fruits were harvested from different citrus species (*C. aurantium*, *C. reshni*, *C. clementina *(var. Clemenules), *C. macrophylla*, and *C. medica*) subjected to different biotic or abiotic stresses or at different developmental stages (Table 1). Adult trees were grown under field conditions at the IVIA (Moncada-Valencia, Spain). Young trees (6-month-old seedlings) were grown in pots (20 cm in diameter × 30 cm in height) in a greenhouse under natural light at 27°C and 60% humidity.

For the water stress treatment, plants were transplanted to pots with dried substrate and tissues were collected at 0, 5, 10 and 24 hours after the treatment. Some of the water stressed plants were watered after 24 hours and tissues were collected 2 and 12 hours later. Salt stressed plants were either watered with 200 mM NaCl (-1.04 MPa) and tissues were collected at 3 hours, 24 hours and 4 days, or were watered with 30 mM (-0.15 MPa) NaCl for 3 months and with 60 mM (-0.32 MPa) NaCl for another 3 months, and tissues were collected when the treatment was brought to completion. For the iron chlorosis assay, plants were watered with an iron scavenger, and foliar tissue was collected from the second, third and fourth bolting after the treatment. For the biotic stresses, plants were infected with *Citrus Tristeza Virus *(CTV), *Citrus Exocortis viroid *(CEVd) or *Hot Stunt Viroid *(HSVd) by grafting plants with bark pieces (containing a stem bud) from infected plants at the IVIA (Instituto Valenciano de Investigaciones Agrarias Moncada, Valencia, Spain), and tissues were collected once the symptoms appeared. For the infection with the oomycete *Phytophthora citrophthora*, the plants were transferred to containers with a solution of zoospores. Once the infection (foot rot) developed, roots were collected. Some postharvest stresses, such as cold stress or the infection with the fungus *Penicillium digitatum*, were assayed in harvested mature fruits. For the cold treatment, fruits were stored at 2°C and the tissues were collected at different times, from hours to several months. Harvested mature fruits were infected with spores of *P. digitatum *following wounding of the fruit surface and kept under high humidity conditions for 8, 12 and 24 hours. For developmental studies, different tissues were taken at various developmental stages: leaves (2-3, 3-4, 4-5, 5-6 and 6-8 cm in length); apical meristems (1-2 cm long); inflorescences (3-4, 5-6 and 8-9 cm in length); unpollinated ovaries (closed floral bud, petal fall, anthesis, and senescent); and fruits (1, 3, 7 and 14 days after treatment of flowers with GA_3_).

Wild-type and transgenic *Arabidopsis thaliana *plants (Col-0) were grown in chambers under 16 h light - 8 h dark regime, at 22°C. Seeds were surface-sterilized and selected in Murashige and Skoog (MS) media.

### RNA isolation for library construction

Total RNA was extracted from different organs and tissues by phenol separation and LiCl precipitation according to Bugos et al. [42] with minor modifications. Poly(A^+^) RNA was isolated from total RNA using Oligotex mRNA kit (Qiagen).

### Development of a Gateway-based cDNA cloning vector

Prior to the construction of the full-length enriched library we developed a cloning vector (pENTR-*Sfi*I) allowing for both effective directional cloning by taking advantage of the nonsymmetrical cleavage of the *Sfi*I restriction enzyme and for the ease of subcloning provided by the Gateway System. For that purpose, we performed several modifications in the commercially available Invitrogen's pENTR1A vector. Briefly, pENTR1A plasmid was digested with *EcoR*I and *Xho*I to remove the *ccdB *gene. The pENTR-*Sfi*I vector was obtained by ligation of a *Sfi*I adaptor, containing two different recognition sequences for the *Sfi*I restriction enzyme (*Sfi*IA and *Sfi*IB, underlined), to the digested vector arms. The adaptor was prepared by annealing the oligonucleotides pENTR-*Sfi*I-F (5'-AATTCGGCCATTATGGCCTGCAGGATCCGGCCGCCTCGGCC-3') and pENTR-*Sfi*I-R (5'-TCGAGGCCGAGGCGGCCGGATCCTGCAGGCCATAATGGCCG-3') after heating for 10 minutes at 70°C and slowly cooling at RT. The *Sfi*I recognition sites in the oligos are underlined. *EcoR*I and *Xho*I sites were restored in the pENTR-*Sfi*I vector. Ligation product was used to transform competent JM110 *E. coli *cells. Although there is no recognition DNA sequence in our *Sfi*I recognition sites for Dam or Dcm methylation, we observed that digestion with *Sfi*I was much more efficient in JM110 cells, which are deficient in Dam and Dcm methylation, than in the conventional DH5α competent cells. Prior to its use as cloning vector, the pENTR-*Sfi*I was digested with *Sfi*I (Roche) for 4 hours at 50°C and dephosphorylated by incubation with shrimp alkaline phosphatase (USB) at 37°C for 90 minutes.

### Construction of full-length enriched cDNA libraries

Additional File 1 outlines the methodology for cDNA construction followed in this work. For the construction of the full-length enriched cDNA libraries we used the SMART™ technology with minor modifications [6]. The BD SMART™ PCR cDNA Synthesis kit (BD Biosciences) was used for the synthesis of cDNA starting from 0.5 μg of poly(A^+^) RNA. The first-strand cDNA synthesis, dC tailing and template switching and extension by RT were performed according to the instructions of the manufacturer. However, the primers used in the reaction were the BD Biosciences' oligonucleotides SMART IV (5'-AAGCAGTGGTATCAACGCAGAGTGGCCATTATGGCCGGG-3'), containing the *Sfi*IA recognition site (underlined), and the CDSIII/3' PCR primer (5'-ATTCTAGAGGCCGAGGCGGCCGACATG-d(T)_30_N_-1_N-3'), containing the *Sfi*IB recognition sequence (underlined). We made 6 second-strand synthesis reactions for every cDNA library. Three reactions were used to generate the full-length cDNA libraries described here and the other three reactions were stored to generate the normalized cDNA libraries described below. For each of the second-strand synthesis reactions, 2 μl of the single stranded cDNA were amplified by PCR in a Perkin Elmer 9400 thermal cycler using the following parameters: an initial preheating at 95°C for 1 min and additional 16 cycles of 5 s at 95°C, 5 s at 65°C and 6 min at 68°C. The PCR mix was in accordance with the manufacturer instructions. Next, the cDNA obtained in every second-strand reaction was blunt-ended following the provided instructions. Once polished, the cDNA was digested with 60 u of *Sfi*I (Roche), purified using the Qiaquick PCR Purification kit (Qiagen), and electrophoresed in a 1× TAE 1% agarose gel. Fragments ranging between 1 kb and 5 kb were purified with the Qiaquick Gel Extraction kit. The ds-cDNA was then ready to be ligated into the modified pENTR-*Sfi*I vector. Ligation was performed with 10 ng of pENTR-*Sfi*I vector and 10 ng of cDNA, with 2 u of T4 DNA ligase (Roche) in a final volume of 10 μl. The reaction was incubated at 16°C for 18 hours. One half of the ligation reaction was used to transform One Shot MAX Efficiency DH5α-T1 Competent Cells (Invitrogen), and transformed cells were selected onto LB agar plates supplemented with 50 μg/ml kanamycin.

### Normalization of cDNA and construction of normalized cDNA libraries

For the normalization we took advantage of the properties of the Duplex-Specific Nuclease (DSN) enzyme purified from Kamchatka crab hepatopancreas (Evrogen) [24]. DSN displays a strong preference for cleaving ds DNA and DNA in DNA-RNA hybrid duplexes. The substrates of the DSN were the three ds-cDNA synthesis reactions left aside during the full-length library construction described above. Two reactions were stored before cDNA was blunt-ended as a control of the normalization and a third reaction was used for normalization.

Most of the normalized cDNA libraries were prepared from the same mixture of ds-cDNA previously produced to obtain the full-length cDNA libraries (Table 1 and Additional File 1). The normalization process was carried out following the protocol of a cDNA Normalization kit provided by Evrogen with some modifications, to make it suitable for the construction of our specific cDNA libraries. Purified ds-cDNA (120 ng) was mixed with 4 μl of 4× hybridization buffer (200 mM Hepes, pH 7.5 and 2 M NaCl), in a 16 μL-final volume, and aliquoted in 4 PCR tubes. The ds-cDNA was denatured by incubating the tubes at 98°C for 2 minutes. Then, re-hybridization was performed by incubation at 68°C for 5 hours. Preheated DSN master buffer (5 μl per tube) was added and tubes were incubated at 68°C for additional 10 minutes. Different enzyme amounts were assayed in each tube (1, 0.5, 0.25 and 0 Kunitz-unit). Degradation reaction was carried out for 25 min at 68°C. To quench the reaction, 10 μl of DSN stop solution were added to each tube followed by incubation at 68°C for 5 minutes. Reaction was finished by cooling down the tubes on ice, and finally 20 μl of water were added to bring the final volume up to 40 μl per tube.

### First run of amplification of the normalized cDNA

The PCR amplifications were performed using the reagents provided in Advantage 2 PCR kit (BD Biosciences). For the first run of amplification we designed new primers suitable for maintaining the average cDNA length. Specifically, their sequences were internal and shorter than those employed in the synthesis of the ds-cDNA. As in the previous normalization step, four PCR amplifications were done individually, each of them containing 1 μl of the normalized cDNA with different concentrations of DSN enzyme obtained in the previous step. The PCR master mix for all the four reaction tubes was prepared by combining the following reagents in the order shown: 39 μl of sterile water, 5 μl of 10× Advantage PCR Buffer, 1 μl of 50× dNTP mix, 1.5 μl of primer M1-5' 10 μM (5'-AAGCAGTGGTATCAACGCAGAGT-3'), 1.5 μl of primer M1-3' 10 μM (5'-ATTCTAGAGGCCGAGGCGG-3') and 1 μl of 50× Advantage Polymerase mix. The thermal cycling reactions were carried out in a preheated (95°C) Perkin-Elmer 9400 machine using the following parameters: 7 sec at 95°C, 10 sec at 66°C and 6 min at 72°C. As the optimal number of cycles had to be established empirically for each cDNA library to be done, a 10 μl aliquot of each PCR reaction was transferred to a clean tube after 7, 9, 11, 13, and 15 PCR cycles. At the end of this process, we obtained a series of 5 tubes from every initial PCR reaction (20 in total).

An aliquot of 5 μl from each tube was electrophoresed in a TAE 1.5× agarose gel to determine the efficiency of normalization. The cDNA used in the next step was chosen according to three characteristics: 1) overall signal intensity of the smear is similar to that shown for the control (without DSN digestion) but does not contain distinguishable bands, 2) signal intensity of smear has reached its plateau, and 3) the cDNA smear does not exceed 4.5 kb.

### Second run of amplification of the normalized cDNA

Both the reaction that best fit in the normalization parameters and the non-normalized reaction (control) were diluted 1:10. A second run of amplification was carried out with an aliquot of 2 μl from those dilutions. To increase the amount of cDNA, three reactions of amplification were performed for the normalized cDNA. The PCR master mix was prepared by combining the following reagents: 76 μl of sterile water, 10 μl of 10× Advantage PCR buffer, 2 μl of 50× dNTP mix, 4 μl of primer M2-5' 10 μM (5'-AAGCAGTGGTATCAACGCAG-3'), 4 μl of primer M2-3' 10 μM (5'-ATTCTAGAGGCCGAGGCG-3') and 2 μl of 50× Advantage Polymerase mix. The thermal cycling reactions were carried out in a preheated (95°C) Perkin-Elmer 9400 machine with 12 cycles of 7 sec at 95°C, 10 sec at 66°C and 6 min at 72°C. Once finished, 300 μl (100 μl × 3 reactions) of normalized cDNA were ready to be used in the construction of the normalized cDNA library.

### Last steps for the construction of the normalized cDNA library

The next steps in the construction of the normalized cDNA library (cDNA polishing, digestion and electrophoresis of digested cDNA, cDNA purification, ligation into pENTR-*Sfi*I vector and transformation of competent *E. coli *cells) were identical to those described above for the construction of the full-length cDNA libraries.

### Virtual northern to assess the normalization efficiency

For a better assessment of the normalization efficiency we performed a virtual northern to estimate the relative concentration of a highly abundant clone in both the non-normalized and the normalized cDNA populations obtained from the second run of amplification. Equivalent quantities of cDNA corresponding to the non-normalized and normalized samples subjected to the second run of amplification were electrophoresed in a TAE 1.5× gel, transferred to a nitrocellulose membrane, followed by a standard Southern blot analysis. A PCR was performed to amplify the C32009H03 clone, which was previously identified as highly abundant in our RVDevelop1 library, using vector oligos (pENTR-F: 5'-GGCTTTAAAGGAACCAATTCA-3' and pENTR-R: 5'-GCAATGCTTTCTTATAATGCCAAC-3'). Ten nanograms of this PCR fragment were used in a labeling reaction with [α-^32^P]dCTP using Ready to Go DNA labeling kit (Amersham Bioscience), and reaction was purified from unincorporated nucleotides using probe purification columns (NucTrap, Stratagene, or Quick Spin, Roche). The DNA blot membrane was hybridized according to the protocol described by Church and Gilbert [43].

### EST collection and sequencing

Colonies were randomly-selected from LB agar plates supplemented with 50 μg/ml kanamycin. After manual picking, colonies were grown overnight in standard LB-kanamycin media, and plasmids were isolated by alkaline lysis method using PerfectPrep (Eppendorf) or Montage (Millipore) kits. In addition, selected clones were stored at -80°C as glycerol stocks. Sequencing reactions were carried out from the 5' end of the cDNA insert, with the pENTR-F oligo (5'-GGCTTTAAAGGAACCAATTCA-3'), using an ABI 3100 capillary automatic sequencer (Applied Biosystems) with fluorescent dye terminator technology.

### EST preprocessing and assembly

EST preprocessing and assembly was performed by using EST2uni, an open, parallel software package for EST analysis and database creation [44]. EST2uni is a completely automated computer pipeline capable of converting, using the standard EST analysis tools described below, a group of input sequence trace files in a highly structured and annotated EST database with a user-oriented web interface for efficient data mining. All of the data generated by the pipeline were stored in a web-searchable MySQL database . Base calling and quality scores assignment were made with Phred [45] using default parameters. Low quality and cloning vector trimming was performed by Lucy [46]. Contaminant vector sequences were detected and removed by SeqClean  using the NCBI vector database UniVec . Repeat elements and low complexity region masking were done with RepeatMasker  and SeqClean , respectively. After cleaning, sequences shorter than 100 nucleotides were discarded. Clean, high-quality EST sequences obtained in the pre-processing step were then assembled in contigs and singletons to obtain a non-redundant unigene set using TGICL  with default parameters. ESTs generated in this study were assembled together with the rest of ESTs obtained in the whole CFGP  in order to improve the quality of the assembly.

To estimate the number of new ESTs and unigenes generated in this work, we compared the new citrus ESTs and unigenes with the 229,570 Citrus ESTs in the HarvEST database, version 1.25, as of June 9th 2009 , using BLASTN and Perl scripts. An EST is considered as new if it has at least 25% of sequence with less than 95% of identity to any other EST or unigene in the HarvEST collection.

### Functional annotation of unigenes

Unigene functional annotation was also done by using the EST2uni software package. As a part of the whole analysis, EST2uni carried out BLASTX against the UniRef90 non-redundant protein database [47] and the full set of Arabidopsis proteins provided by The Arabidopsis Information Resource (TAIR)  using default parameters and arbitrary non-stringent threshold of 10^-5 ^for E-value. Unigenes were annotated with the description of the most similar UniRef90 cluster of proteins or, when no significantly similar UniRef90 cluster was found, with the description of the most similar Arabidopsis protein, if any. Unigenes were annotated as highly similar to the first BLAST hit when the E-value was lower than 10^-15^. BLASTX hits with an E-value higher than 10^-10 ^were not considered for annotation. Gene Ontology (GO) annotation of the Arabidopsis most similar protein was used for GO annotation of the citrus unigenes. Functional motifs were also identified by using a HMMER search [48] against the Pfam database [49].

### Isolation and sequencing of a full-length *CitrSEP *clone

Clone C32006D10 (*CitrSEP*), with homology to the Arabidopsis gene *SEP3 *of the MADS-box gene family, was selected. An aliquot (1 μl) of the DNA prep was subjected to PCR amplification using the vector oligos pENTR-F and pENTR-R (see above), and the PCR product was fully sequenced using internal oligos.

### Cloning into Gateway destination vectors

The transfer of the cDNA clone *CitrSEP *from the pENTR-*Sfi*I to the Gateway destination vector pMDC32 was performed using the LR clonase kit (Invitrogen). The reaction mix was prepared by combining 2 μg of plasmid C32006D10, 2 μg of pMDC32 plasmid, 4 μl of 5× LR clonase and 8 μl of reaction buffer, and the sample was then incubated at 25°C for 60 min. The reaction was then treated with 2 μl of proteinase K by incubation at 37°C for 10 minutes. An aliquot (1 μl) from the recombination reaction was used to transform One Shot MAX Efficiency DH5α-T1 Competent Cells (Invitrogen) according to the manufacturer instructions, and cells were selected on LB agar plates containing 50 μg/ml of kanamycin. Selection of positive clones was carried out by PCR analysis.

### Transformation of Arabidopsis plants

The binary plasmid pMDC32 carrying the citrus clone C32006D10 was used to transform *Agrobacterium tumefaciens *GV3101 C58C1 Rifr (pMP90) [50] by electroporation using a Gene-Pulse apparatus (Bio-Rad). Col-0 Arabidopsis plants were transformed by the floral dip method [51]. T1 seeds were plated on MS media supplemented with 50 μg/mL hygromycin for selection of transformants and 500 μg/mL vancomycin to limit the growth of *Agrobacterium*. T2 seeds were collected and homozygous lines were selected based on hygromycin resistance of the T3 progeny. Expression of the *CitrSEP *gene in seedlings was assayed by semi-quantitative and quantitative qRT-PCR, and homozygous lines showing highest *CitrSEP *expression were selected and used for functional characterization.

### Semi-quantitative RT-PCR and qRT-PCR analysis of gene expression

Total RNA was extracted from frozen rosette leaves using the RNeasy Plant Mini kit (Qiagen). Genomic DNA was eliminated during RNA purification by treatment with 50 units of DNaseI (RNase-free DNase set (Qiagen) for 15 min at room temperature. Two micrograms of total RNA were used to synthesize first strand cDNA, using the SuperScript™ First-Strand Synthesis System for RT-PCR (Invitrogen Life Technologies). cDNA synthesis reactions were finally diluted in a volume of 80 μL.

For semi-quantitative RT-PCR, the *ELONGATION FACTOR 1-α *(*EF-1-α*) gene (*At1g07940*) was used as an internal control for quantification. Primers were used to amplify a 532 bp fragment from *EF-1-α *cDNA and a 535 bp fragment from *CitrSEP *cDNA. cDNA solution (1 μL) was used in PCR reactions (50 μL final volume) in the presence of 0.6 μM of each clone-specific primer and 2.6 units of Expand High Fidelity enzyme (Roche). PCR reaction conditions were 5 min at 95°C, followed by 25 cycles of 30 s at 95°C, 45 s at 60°C and 45 s at 72°C, and a final extension of 10 min at 72°C. The products were detected in a 1% (v/v) agarose 1 × TAE gel and stained with ethidium bromide before quantifying using the Gene Snap (SynGene) program.

qRT-PCR was carried out using the SYBR^® ^GREEN PCR Master mix (Applied Biosystems) in an ABI PRISM 7000 Sequence Detection System (Applied Biosystems). Final reaction volume was 20 μL, with 1 μL of cDNA, 10 μL of SYBR^® ^GREEN PCR Master mix, and 9 μL of primer mixture, containing 0.66 μM of each primer. The PCR program consisted of an initial incubation of 2 min at 50°C followed by a de-naturation at 95°C for 10 min, and 40 cycles of amplification of 15 seconds at 95°C and 1 min at 60°C. Each sample was assayed in triplicates, and each experiment was repeated at least twice. Expression levels were calculated relative to the constitutively expressed gene *EF-1-α*. Normalization was carried out using the ΔΔCt method (Applied Biosystems), where ΔCt was calculated for each sample as the difference between Ct of the *CitrSEP *gene and Ct of *EF-1-α*, and final relative expression level was determined as inverse of log_2 _of Ct (*SEP*) - Ct (*EF-1-α*). Primers used are listed in Additional File 8.

## Authors' contributions

MCM set-up the protocols for library construction, cDNA cloning and sequencing, generated the cloning vector, and performed most of the cDNA library construction. MCM, RA, and SA carried out cDNA clone isolation and sequencing. RA and HA carried out Arabidopsis transformation and isolation of homozygous transgenic lines. HA performed all phenotypic and molecular analysis of transgenic plants. JF performed the bioinformatics work. VC directed the Spanish Citrus Genomics Project. MAPA conceived the work. MAPA and MCM wrote the manuscript. All authors read and approved this manuscript.

## Supplementary Material

Additional File 1 **Scheme for the synthesis of cDNA and generation of full-length and normalized libraries**. This file contains a figure schematically showing the approach employed in this study for the generation of citrus normalized full-length cDNA libraries, combining the SMART™ method to generate full-length cDNAs, the thermostable enzyme DSN to normalize cDNA populations, and the Gateway technology. Click here for file

Additional File 2 **Analysis of ESTs**. This file contains figures showing details about the ESTs obtained. **A**, Distribution of EST length. **B**, Distribution of EST number per contig. Click here for file

Additional File 3 **List of the new 2113 ESTs, corresponding to 1831 new unigenes, generated**. The table contains a list of the new ESTs generated not previously included in HarvEST. The corresponding unigenes and the three Gene Ontology classifications (Biological Process, Cellular Component, and Molecular Function) are indicated. Click here for file

Additional File 4 **Distribution of citrus and Arabidopsis genes according to the three GO categories**. This file contains a table showing percentages of genes in the different categories of the three Gene Ontology classifications (Biological Process, Cellular Component, and Molecular Function). Citrus genes are those reported in this work, and Arabidopsis genes are those from TAIR. Click here for file

Additional File 5 **Evaluation of the efficiency of normalization of cDNA libraries**. This file contains figures showing results demonstrating the efficiency of the approach used for normalization of cDNA libraries. A, Gel electrophoresis analysis of 5 μl aliquots from the first amplification of the normalized cDNA taken at different PCR cycles. B, Gel electrophoresis analysis of the normalized cDNA utilized in the construction of the normalized cDNA library RVDevelopN. **C**, Gel electrophoresis analysis of a non-normalized cDNA population (left) and a normalized cDNA population (right) from the same RNA sample. **D**, Virtual northern of the cDNA smear blotted and hybridized with the highly abundant clone C32009H03. Click here for file

Additional File 6 **Sequence of the full-length clone C32006D10**. This file contains cDNA and deduced amino acid sequence of the full-length clone C32006D10 from the cDNA library RVDevelop1 encoding a MADS-box gene *CitrSEP *from citrus, a homologue of the *Arabidopsis AGL9 *or *SEP3*(At1g24260). Click here for file

Additional File 7 **Expression analysis of transgenic Arabidopsis plants that overexpress the *CitrSEP *gene**. This file shows expression of *CitrSEP *and four endogenous *SEPALLATA *genes analyzed by qRT-PCR. Expression was normalized to the expression of the constitutive *EF-1-α *gene and then to the expression in Col-0 plants. For normalization purposes, the detection level of our qRT-PCR analysis was used as an estimate of the *CitrSEP *expression in Col-0. Expression level is indicated in the plot. *nd*, not detected. Click here for file

Additional File 8 **Oligonucleotides used for gene expression by RT-PCR**. This file contains a table with the sequence of oligonucleotides used for RT-PCR assays. Click here for file
