# Supplementary material for: A new set of ESTs and cDNA clones from full-length and normalized libraries for gene discovery and functional characterization in citrus
Source: BMC Genomics. 2009 Sep 11;10:428. doi: 10.1186/1471-2164-10-428 (PMC2754500; doi:10.1186/1471-2164-10-428)
Supplement: Additional File 1 — Scheme for the synthesis of cDNA and generation of full-length and normalized libraries. This file contains a figure schematically showing the approach employed in this study for the generation of citrus normalized full-length cDNA libraries, combining the SMART™ method to generate full-length cDNAs, the thermostable enzyme DSN to normalize cDNA populations, and the Gateway technology. [file 1471-2164-10-428-S1.pdf]

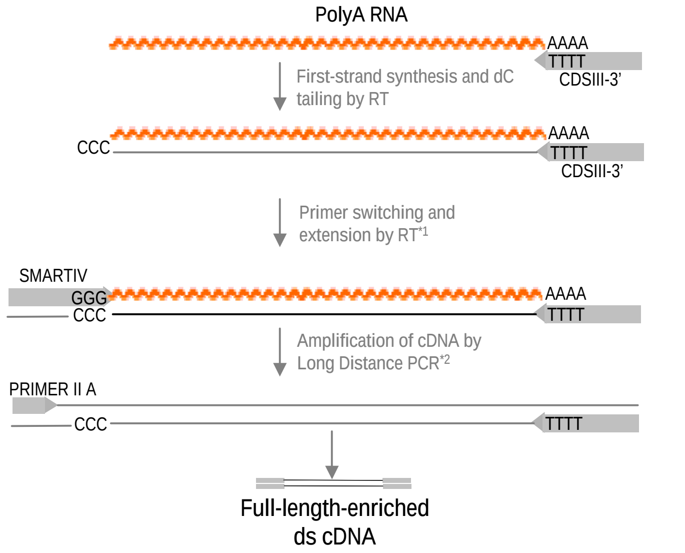

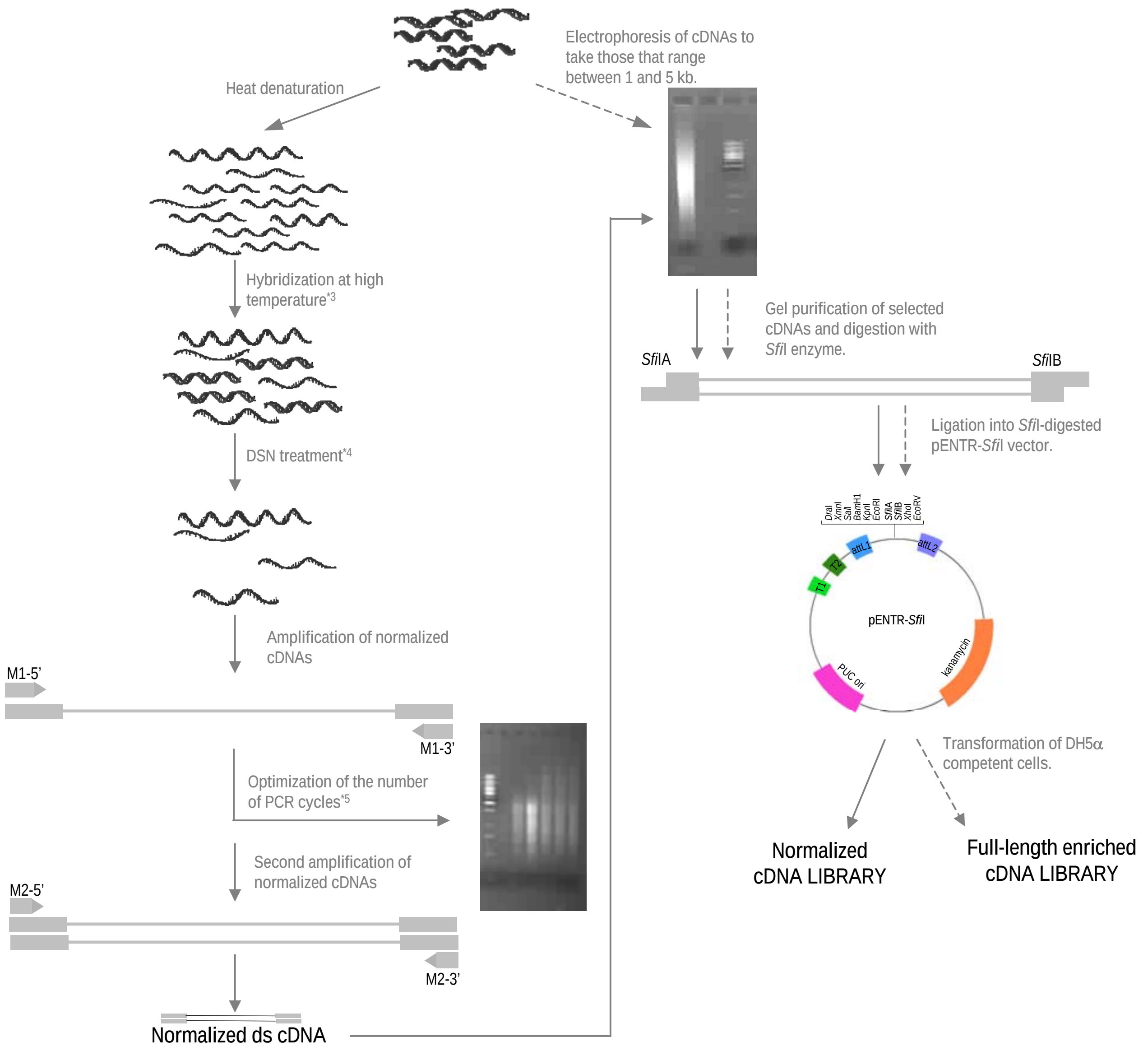

*1 Primer switching is more efficient in full-length cDNAs.

*2 Long-distance PCR allows cloning of long transcripts.

*3 The hybridization step is performed at high temperature (68ºC) for several hours in order to favour the hybridization of abundant transcripts.

*4 DSN treatment must be optimized for every cDNA sample. Run in parallel digestions with different DSN concentration and also a control treatment (without DSN) to compare the results.

*5 Optimization of the number of cycles must be performed. Insuficient number of cycles may result in low cDNA concentration, and excessive number of cycles may render quimeric cDNAs. For that purpose aliquots of the first amplification of the normalized cDNAs taket at different number of cycles must be electrophoresed. The second run of amplification must be performed over the most appropriate population of cDNAs.
