# Supplementary material for: A new set of ESTs and cDNA clones from full-length and normalized libraries for gene discovery and functional characterization in citrus
Source: BMC Genomics. 2009 Sep 11;10:428. doi: 10.1186/1471-2164-10-428 (PMC2754500; doi:10.1186/1471-2164-10-428)
Supplement: Additional File 2 — Analysis of ESTs. This file contains figures showing details about the ESTs obtained. A, Distribution of EST length. B, Distribution of EST number per contig. [file 1471-2164-10-428-S2.ppt]

## Slide 1
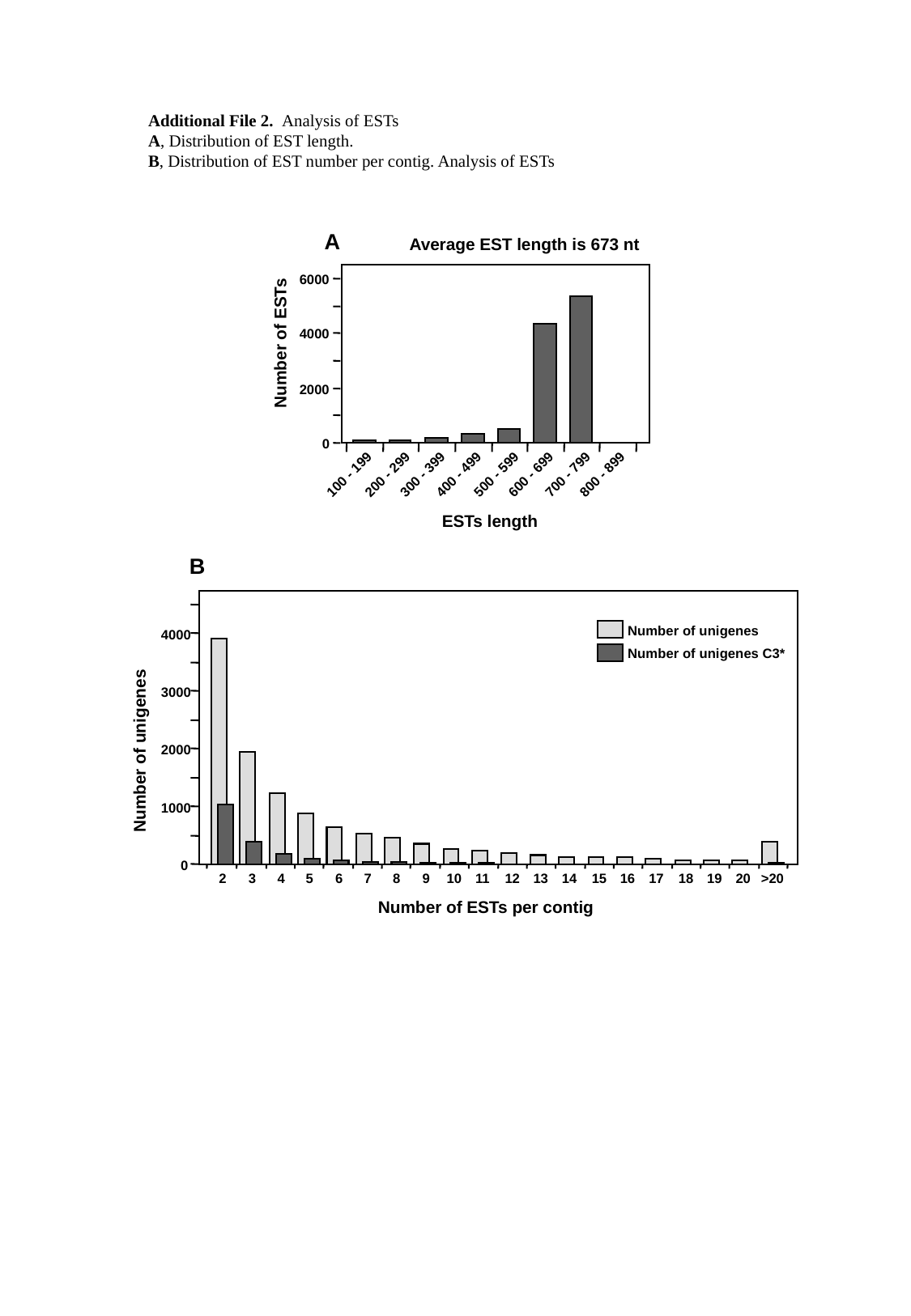

Additional File 2. Analysis of ESTs
A, Distribution of EST length.
B, Distribution of EST number per contig. Analysis of ESTs
A
Average EST length is 673 nt
6000
4000
Number of ESTs
2000
0
100 - 199
200 - 299
300 - 399
400 - 499
500 - 599
600 - 699
700 - 799
800 - 899
ESTs length
B
Number of unigenes
4000
Number of unigenes C3*
3000
2000
1000
0
2
3
4
5
6
7
8
9
10
11
12
13
14
15
16
17
18
19
20
>20
Number of unigenes
Number of ESTs per contig
