# Supplementary material for: A new set of ESTs and cDNA clones from full-length and normalized libraries for gene discovery and functional characterization in citrus
Source: BMC Genomics. 2009 Sep 11;10:428. doi: 10.1186/1471-2164-10-428 (PMC2754500; doi:10.1186/1471-2164-10-428)
Supplement: Additional File 4 — Distribution of citrus and Arabidopsis genes according to the three GO categories. This file contains a table showing percentages of genes in the different categories of the three Gene Ontology classifications (Biological Process, Cellular Component, and Molecular Function). Citrus genes are those reported in this work, and Arabidopsis genes are those from TAIR. [file 1471-2164-10-428-S4.doc]

| **Supplemental File 4.** Distribution of citrus and Arabidopsis genes (total number and percentage) according to the GO categories of Biological process, Cellular component, and Molecular function. Citrus genes are those reported here and Arabidopsis genes are those from TAIR. | | | | |
| --- | --- | --- | --- | --- |
| **GO category** | **CFGP** | | **TAIR** | |
|  | **Genes** | % | **Genes** | % |
| **GO:0008150 Biological Process** | 2314 | 56.92 | 25646 | 60.51 |
| GO:0008152 metabolic process | 1800 | 44.28 | 9809 | 23.14 |
| GO:0009987 cellular process | 1774 | 43.64 | 10673 | 25.18 |
| GO:0019538 protein metabolic process | 750 | 18.45 | 3753 | 8.85 |
| GO:0009058 biosynthetic process | 682 | 16.78 | 4478 | 10.57 |
| GO:0006810 transport | 342 | 8.41 | 1877 | 4.43 |
| GO:0006139 nucleobase, nucleoside, nucleotide and nucleic acid metabolic process | 318 | 7.82 | 2802 | 6.61 |
| GO:0006464 protein modification process | 263 | 6.47 | 1485 | 3.5 |
| GO:0006412 translation | 223 | 5.49 | 1301 | 3.07 |
| GO:0005975 carbohydrate metabolic process | 186 | 4.58 | 847 | 2.00 |
| GO:0009056 catabolic process | 174 | 4.28 | 636 | 1.50 |
| GO:0006350 transcription | 166 | 4.08 | 1786 | 4.21 |
| GO:0006519 amino acid and derivative metabolic process | 160 | 3.94 | 511 | 1.21 |
| GO:0006950 response to stress | 155 | 3.81 | 2023 | 4.77 |
| GO:0016043 cellular component organization and biogenesis | 120 | 2.95 | 1293 | 3.05 |
| GO:0006629 lipid metabolic process | 96 | 2.36 | 727 | 1.72 |
| GO:0007154 cell communication | 93 | 2.29 | 1161 | 2.74 |
| GO:0006091 generation of precursor metabolites and energy | 91 | 2.24 | 221 | 0.52 |
| GO:0007165 signal transduction | 90 | 2.21 | 1021 | 2.41 |
| GO:0019748 secondary metabolic process | 78 | 1.92 | 351 | 0.83 |
| GO:0009628 response to abiotic stimulus | 63 | 1.55 | 1184 | 2.79 |
| GO:0015979 photosynthesis | 43 | 1.06 | 147 | 0.35 |
| GO:0009719 response to endogenous stimulus | 38 | 0.93 | 805 | 1.9 |
| GO:0008219 cell death | 29 | 0.71 | 140 | 0.33 |
| GO:0016265 death | 29 | 0.71 | 140 | 0.33 |
| GO:0009605 response to external stimulus | 29 | 0.71 | 282 | 0.67 |
| GO:0007275 multicellular organismal development | 26 | 0.64 | 1705 | 4.02 |
| GO:0006259 DNA metabolic process | 23 | 0.57 | 331 | 0.78 |
| GO:0009607 response to biotic stimulus | 20 | 0.49 | 634 | 1.50 |
| GO:0000003 reproduction | 14 | 0.34 | 1009 | 2.38 |
| GO:0019725 cellular homeostasis | 12 | 0.30 | 159 | 0.38 |
| GO:0009653 anatomical structure morphogenesis | 10 | 0.25 | 412 | 0.97 |
| GO:0040007 growth | 7 | 0.17 | 267 | 0.63 |
| GO:0030154 cell differentiation | 6 | 0.15 | 284 | 0.67 |
| GO:0009791 post-embryonic development | 6 | 0.15 | 509 | 1.20 |
| GO:0016049 cell growth | 5 | 0.12 | 219 | 0.52 |
| GO:0022904 respiratory electron transport chain | 5 | 0.12 | 29 | 0.07 |
| GO:0009790 embryonic development | 4 | 0.10 | 522 | 1.23 |
| GO:0007049 cell cycle | 3 | 0.07 | 213 | 0.50 |
| GO:0009908 flower development | 3 | 0.07 | 270 | 0.64 |
| GO:0009835 ripening | 2 | 0.05 | 3 | 0.01 |
| GO:0009991 response to extracellular stimulus | 2 | 0.05 | 69 | 0.16 |
| GO:0040029 regulation of gene expression, epigenetic | 1 | 0.02 | 150 | 0.35 |
| GO:0007610 behavior | 0 | 0.00 | 6 | 0.01 |
| GO:0007267 cell-cell signaling | 0 | 0.00 | 42 | 0.10 |
| GO:0009875 pollen-pistil interaction | 0 | 0.00 | 42 | 0.10 |
| GO:0032543 mitochondrial translation | 0 | 0.00 | 0 | 0.00 |
| GO:0032544 plastid translation | 0 | 0.00 | 1 | 0.00 |
| GO:0009838 abscission | 0 | 0.00 | 13 | 0.03 |
| GO:0009856 pollination | 0 | 0.00 | 98 | 0.23 |
| GO:0009606 tropism | 0 | 0.00 | 50 | 0.12 |
| **GO:0005575 Cellular Component** | 2672 | 65.73 | 27712 | 65.38 |
| GO:0005623 cell | 2670 | 65.68 | 19296 | 45.53 |
| GO:0005622 intracellular | 1901 | 46.77 | 11296 | 26.65 |
| GO:0005737 cytoplasm | 1607 | 39.53 | 8107 | 19.13 |
| GO:0016020 membrane | 967 | 23.79 | 5997 | 14.15 |
| GO:0009536 plastid | 725 | 17.84 | 4430 | 10.45 |
| GO:0005739 mitochondrion | 592 | 14.56 | 1320 | 3.11 |
| GO:0005634 nucleus | 268 | 6.59 | 2725 | 6.43 |
| GO:0005840 ribosome | 116 | 2.85 | 663 | 1.56 |
| GO:0005829 cytosol | 51 | 1.25 | 706 | 1.67 |
| GO:0005886 plasma membrane | 34 | 0.84 | 2711 | 6.40 |
| GO:0009579 thylakoid | 33 | 0.81 | 786 | 1.85 |
| GO:0005783 endoplasmic reticulum | 25 | 0.62 | 343 | 0.81 |
| GO:0005794 Golgi apparatus | 19 | 0.47 | 226 | 0.53 |
| GO:0005856 cytoskeleton | 14 | 0.34 | 187 | 0.44 |
| GO:0005730 nucleolus | 12 | 0.3 | 348 | 0.82 |
| GO:0030312 external encapsulating structure | 11 | 0.27 | 690 | 1.63 |
| GO:0005618 cell wall | 11 | 0.27 | 686 | 1.62 |
| GO:0005576 extracellular region | 9 | 0.22 | 514 | 1.21 |
| GO:0005777 peroxisome | 6 | 0.15 | 272 | 0.64 |
| GO:0005773 vacuole | 4 | 0.10 | 746 | 1.76 |
| GO:0005654 nucleoplasm | 4 | 0.10 | 130 | 0.31 |
| GO:0005635 nuclear envelope | 3 | 0.07 | 57 | 0.13 |
| GO:0005768 endosome | 2 | 0.05 | 48 | 0.11 |
| GO:0005578 proteinaceous extracellular matrix | 2 | 0.05 | 0 | 0.00 |
| GO:0005764 lysosome | 0 | 0.00 | 3 | 0.01 |
| GO:0005615 extracellular space | 0 | 0.00 | 4 | 0.01 |
| **GO:0003674 Molecular Function** | 3123 | 76.83 | 26204 | 61.83 |
| GO:0003824 catalytic activity | 1853 | 45.58 | 7694 | 18.15 |
| GO:0005488 binding | 1333 | 32.79 | 9387 | 22.15 |
| GO:0016787 hydrolase activity | 583 | 14.34 | 2649 | 6.25 |
| GO:0016740 transferase activity | 510 | 12.55 | 2662 | 6.28 |
| GO:0003676 nucleic acid binding | 465 | 11.44 | 3817 | 9.01 |
| GO:0000166 nucleotide binding | 410 | 10.09 | 1363 | 3.22 |
| GO:0005215 transporter activity | 264 | 6.49 | 1262 | 2.98 |
| GO:0005515 protein binding | 262 | 6.45 | 2859 | 6.75 |
| GO:0003677 DNA binding | 226 | 5.56 | 2261 | 5.33 |
| GO:0016301 kinase activity | 216 | 5.31 | 1349 | 3.18 |
| GO:0030528 transcription regulator activity | 185 | 4.55 | 1896 | 4.47 |
| GO:0005198 structural molecule activity | 182 | 4.48 | 544 | 1.28 |
| GO:0003700 transcription factor activity | 147 | 3.62 | 1682 | 3.97 |
| GO:0003723 RNA binding | 113 | 2.78 | 1110 | 2.62 |
| GO:0008135 translation factor activity, nucleic acid binding | 58 | 1.43 | 135 | 0.32 |
| GO:0045182 translation regulator activity | 58 | 1.43 | 136 | 0.32 |
| GO:0030234 enzyme regulator activity | 49 | 1.21 | 295 | 0.70 |
| GO:0019825 oxygen binding | 36 | 0.89 | 231 | 0.55 |
| GO:0004871 signal transducer activity | 32 | 0.79 | 385 | 0.91 |
| GO:0004518 nuclease activity | 31 | 0.76 | 158 | 0.37 |
| GO:0008289 lipid binding | 18 | 0.44 | 172 | 0.41 |
| GO:0030246 carbohydrate binding | 10 | 0.25 | 116 | 0.27 |
| GO:0004872 receptor activity | 9 | 0.22 | 206 | 0.49 |
| GO:0003774 motor activity | 5 | 0.12 | 87 | 0.21 |
| GO:0003682 chromatin binding | 3 | 0.07 | 31 | 0.07 |
| GO:0005102 receptor binding | 0 | 0.00 | 41 | 0.10 |
