# Supplementary material for: A new set of ESTs and cDNA clones from full-length and normalized libraries for gene discovery and functional characterization in citrus
Source: BMC Genomics. 2009 Sep 11;10:428. doi: 10.1186/1471-2164-10-428 (PMC2754500; doi:10.1186/1471-2164-10-428)
Supplement: Additional File 5 — Evaluation of the efficiency of normalization of cDNA libraries. This file contains figures showing results demonstrating the efficiency of the approach used for normalization of cDNA libraries. A, Gel electrophoresis analysis of 5 μl aliquots from the first amplification of the normalized cDNA taken at different PCR cycles. B, Gel electrophoresis analysis of the normalized cDNA utilized in the construction of the normalized cDNA library RVDevelopN. C, Gel electrophoresis analysis of a non-normalized cDNA population (left) and a normalized cDNA population (right) from the same RNA sample. D, Virtual northern of the cDNA smear blotted and hybridized with the highly abundant clone C32009H03. [file 1471-2164-10-428-S5.ppt]

## Slide 1
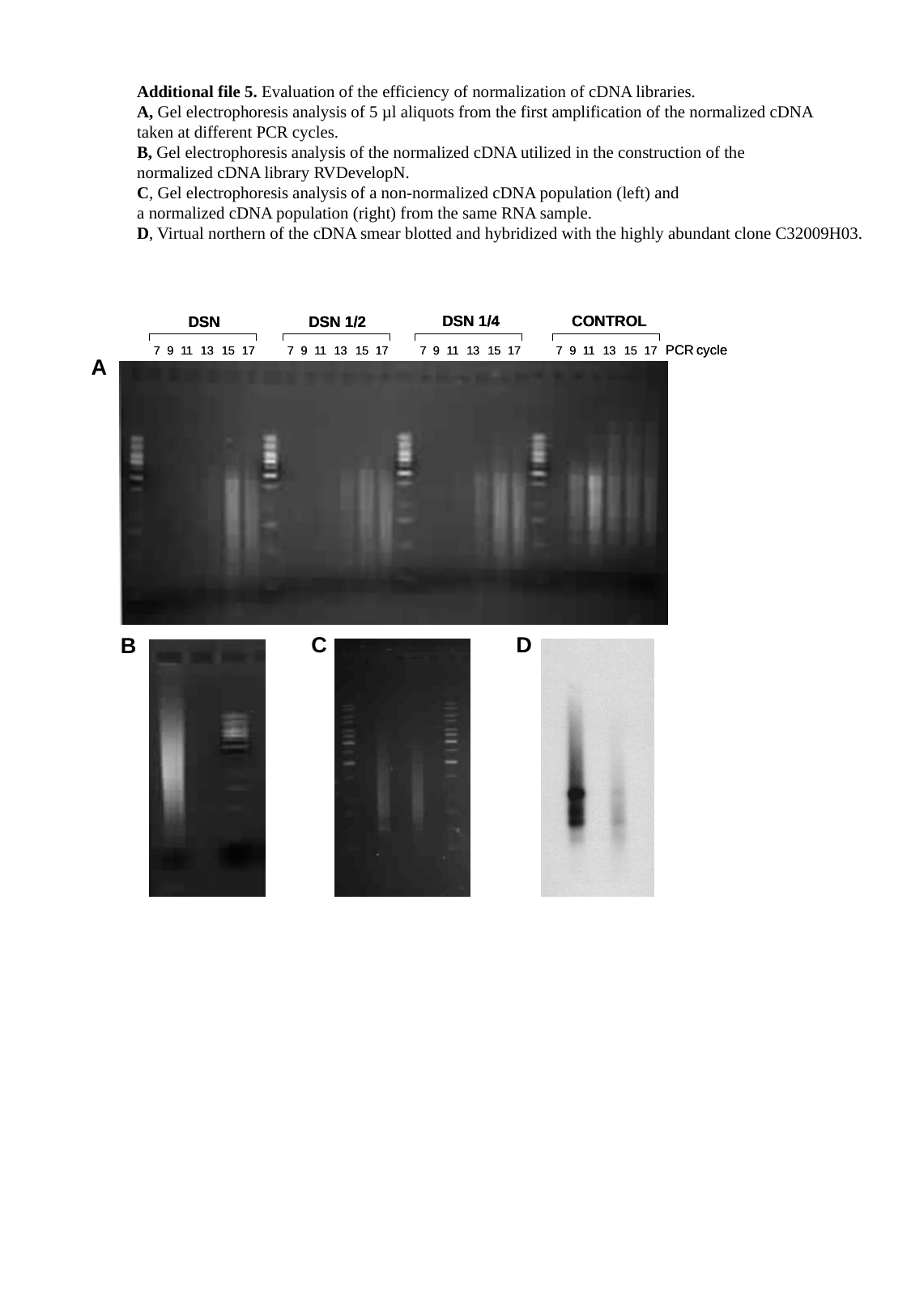

Additional file 5. Evaluation of the efficiency of normalization of cDNA libraries.
A, Gel electrophoresis analysis of 5 µl aliquots from the first amplification of the normalized cDNA
taken at different PCR cycles.
B, Gel electrophoresis analysis of the normalized cDNA utilized in the construction of the
normalized cDNA library RVDevelopN.
C, Gel electrophoresis analysis of a non-normalized cDNA population (left) and
a normalized cDNA population (right) from the same RNA sample.
D, Virtual northern of the cDNA smear blotted and hybridized with the highly abundant clone C32009H03.
DSN 1/4
DSN 1/4
CONTROL
CONTROL
DSN
DSN
DSN 1/2
DSN 1/2
PCR
PCR
cycle
cycle
7
7
9
9
11
11
13
13
15
15
17
17
7
7
9
9
11
11
13
13
15
15
17
17
7
7
9
9
11
11
13
13
15
15
17
17
7
7
9
9
11
11
13
13
15
15
17
17
A
C
D
B
