# Supplementary material for: A new set of ESTs and cDNA clones from full-length and normalized libraries for gene discovery and functional characterization in citrus
Source: BMC Genomics. 2009 Sep 11;10:428. doi: 10.1186/1471-2164-10-428 (PMC2754500; doi:10.1186/1471-2164-10-428)
Supplement: Additional File 6 — Sequence of the full-length clone C32006D10. This file contains cDNA and deduced amino acid sequence of the full-length clone C32006D10 from the cDNA library RVDevelop1 encoding a MADS-box gene CitrSEP from citrus, a homologue of the Arabidopsis AGL9 or SEP3(At1g24260). [file 1471-2164-10-428-S6.doc]

### Additional file 6. Sequence of the full-length clone C32006D10.

cDNA and deduced amino acid sequence of the full-length clone C32006D10 from the cDNA library RVDevelop1 encoding a MADS-box gene *CitrSEP* from citrus, a homologue of the *Arabidopsis* *AGL9* or *SEP3* (At1g24260).

ggagaaagaaaggagaagaaaatgggaaggggtagggttgagttgaagaggatagagaac

M G R G R V E L K R I E N

aagatcaacaggcaagtgacctttgcaaagagaagaaatggccttttgaagaaagcttat

K I N R Q V T F A K R R N G L L K K A Y

gagctttccgttctttgtgatgctgaggttgctctcatcattttctccaatagaggaaag

E L S V L C D A E V A L I I F S N R G K

ctgtacgagttttgcagtagttcaagcatgctcaaaacgcttgagaggtaccagaagtgc

L Y E F C S S S S M L K T L E R Y Q K C

aactatggagcaccagaaccgaatgtgtccgcaagggaggccctggagctaagtagccag

N Y G A P E P N V S A R E A L E L S S Q

caagaatatctgaagcttaaagcacgatatgaagccctacagagatcccaaaggaatctc

Q E Y L K L K A R Y E A L Q R S Q R N L

cttggagaagaactcggccctctaaacagcaaagagcttgagtcacttgaaaggcagctt

L G E E L G P L N S K E L E S L E R Q L

gatatgtcattgaagcagatcagatcaacaagaactcagtacatgctggatacccttact

D M S L K Q I R S T R T Q Y M L D T L T

gaactgcaacataaggaacagttgctgagcgaagcaaataagaccctcaaacaaaggttg

E L Q H K E Q L L S E A N K T L K Q R L

atggagggataccaagtgaacacacttcaattgaatcctagtgcagaagattgtggttat

M E G Y Q V N T L Q L N P S A E D C G Y

gggcttaaaccagctcaacctcagggcgataccttctttcacgccttggaatgtgaaccc

G L K P A Q P Q G D T F F H A L E C E P

acattgcaaattggataccagcctgcggacccaatatcggttgtcacagcaggcccgagt

T L Q I G Y Q P A D P I S V V T A G P S

ctgaataattacatgcaaggatggctaccatgctaagagagcggcgggcattaccagtca

L N N Y M Q G W L P C *

atgtattgcagttgatccataagaatcagcaactttgtatccctttgtaagcaaccctat

tcagtgactaggaacacgcaatcataaggttttattataataactctacagtgatcctgc

actttagggaaactgatgacgatatatagctatgttgtgtgtgagtgtattttcaatcac

cgcgcttatctttatttaacaaagccttgtttgagaacc
