# Supplementary material for: A new set of ESTs and cDNA clones from full-length and normalized libraries for gene discovery and functional characterization in citrus
Source: BMC Genomics. 2009 Sep 11;10:428. doi: 10.1186/1471-2164-10-428 (PMC2754500; doi:10.1186/1471-2164-10-428)
Supplement: Additional File 7 — Expression analysis of transgenic Arabidopsis plants that overexpress the CitrSEP gene. This file shows expression of CitrSEP and four endogenous SEPALLATA genes analyzed by qRT-PCR. Expression was normalized to the expression of the constitutive EF-1-α gene and then to the expression in Col-0 plants. For normalization purposes, the detection level of our qRT-PCR analysis was used as an estimate of the CitrSEP expression in Col-0. Expression level is indicated in the plot. nd, not detected. [file 1471-2164-10-428-S7.ppt]

## Slide 1
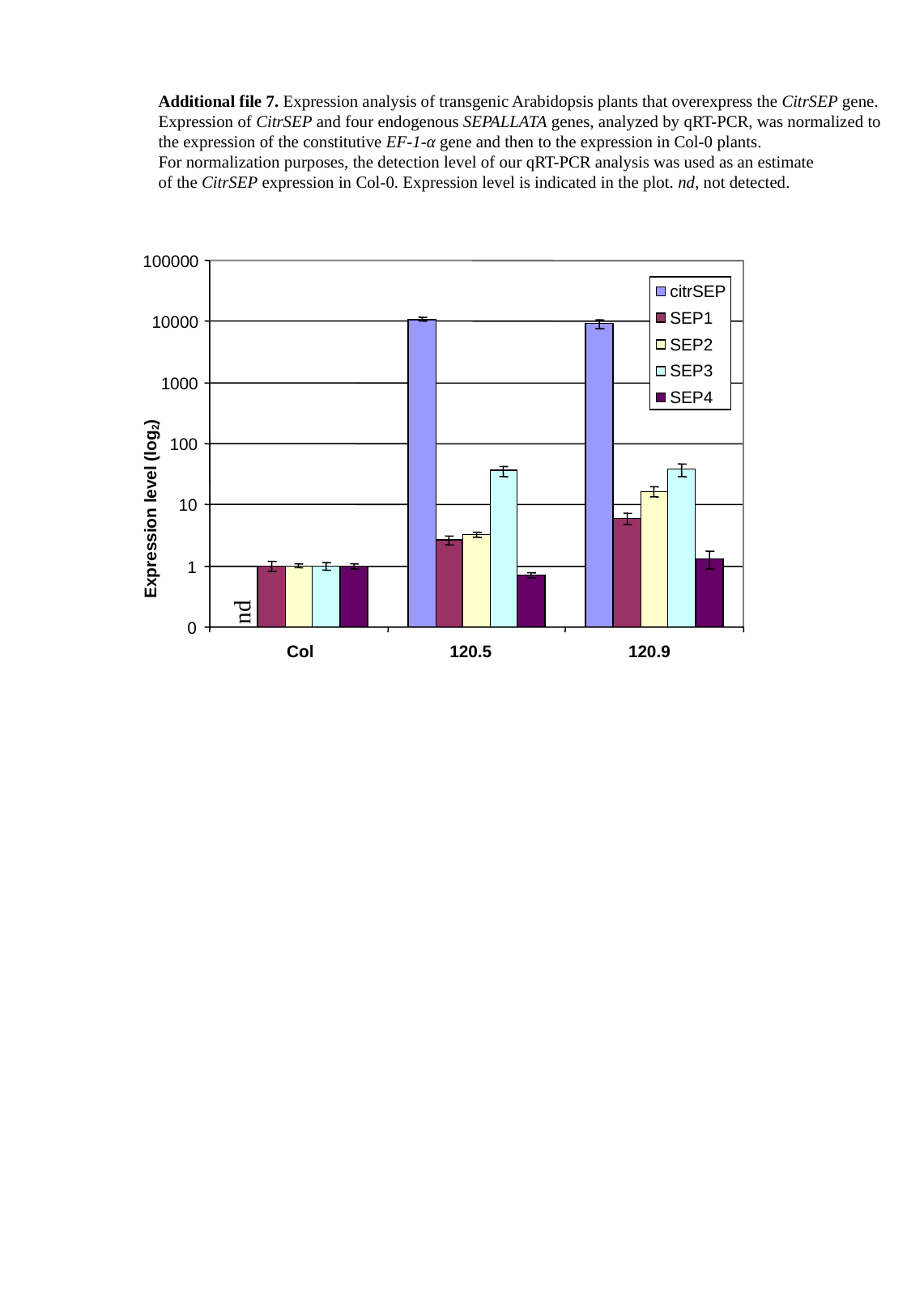

Additional file 7. Expression analysis of transgenic Arabidopsis plants that overexpress the CitrSEP gene.
Expression of CitrSEP and four endogenous SEPALLATA genes, analyzed by qRT-PCR, was normalized to
the expression of the constitutive EF-1-α gene and then to the expression in Col-0 plants.
For normalization purposes, the detection level of our qRT-PCR analysis was used as an estimate
of the CitrSEP expression in Col-0. Expression level is indicated in the plot. nd, not detected.
100000
citrSEP
SEP1
10000
SEP2
SEP3
1000
SEP4
100
10
Expression level (log2)‏
1
nd
0
Col
120.5
120.9
