# Supplementary material for: A new set of ESTs and cDNA clones from full-length and normalized libraries for gene discovery and functional characterization in citrus
Source: BMC Genomics. 2009 Sep 11;10:428. doi: 10.1186/1471-2164-10-428 (PMC2754500; doi:10.1186/1471-2164-10-428)
Supplement: Additional File 8 — Oligonucleotides used for gene expression by RT-PCR. This file contains a table with the sequence of oligonucleotides used for RT-PCR assays. [file 1471-2164-10-428-S8.doc]

| **Supplemental File 8.** Oligonucleotides used for gene expression by RT-PCR. | | |
| --- | --- | --- |
| Primer name | Sequence (5’---3’) | Use and gene |
| EF1α-F | CCCAGGCTGATTGTGCTGT | qRT-PCR  internal control *EF1-α* |
| EF1α-R | GGGTAGTGGCATCCATCTTGTT |
| QRT_athSEP1-F | ATAATGGTCCTGAGGATCTTCAAGA | qRT-PCR  *AtSEP1* |
| QRT_athSEP1-R | GCAAGGATTCCCAAAAGATACAA |
| QRT_athSEP2-F | GCGATACTTCTTCCCCCAATAA | qRT-PCR  *AtSEP2* |
| QRT_athSEP2-R | AAGACCCCACCAGTACTTGCTTAA |
| QRT_athSEP3-2F | CCCTCTCTTTTTTTTTTGACATTTTTAA | qRT-PCR  *AtSEP3* |
| QRT_athSEP3-2R | ACATGAGAGAGGTAATAAAATAGAAACATCA |
| QRT_athSEP4-F | AGGAGAAAGTTGGAGGACAGTGA | qRT-PCR  *AtSEP4* |
| QRT_athSEP4-R | TCCCCAAAACGATTGAGTAAGTG |
| QRT_SEP-F | TGAAGCCCTACAGAGATCCCAA | qQRT-PCR  *citrSEP* |
| QRT_SEP-R | GGGCCGAGTTCTTCTCCAAG |
| EF1α-1F | GCTTTACTCTCTTGCGATATCTCTG | semiqRT-PCR  internal control *EF1-α* |
| EF1α-1R | CTTGGGGGTAGTGGCATCC |
| RT_SEP-F | TGAAGCCCTACAGAGATCCCAA | semiqRT-PCR  *CitrSEP* |
| citrSEP-2R | TGATTGCGTGTTCCTAGTCACTG |
| qRT-PCR, quantitative Real Time PCR  semiqRT-PCR, semi-quantitative Real Time PCR | | |
